# A Comparison of Methods for Analyzing Viral Load Data in Studies of HIV Patients

**DOI:** 10.1371/journal.pone.0130090

**Published:** 2015-06-19

**Authors:** Charles E. Rose, Lytt Gardner, Jason Craw, Sonali Girde, Andrew J. Wawrzyniak, Mari-Lynn Drainoni, Jessica Davila, Jack DeHovitz, Jeanne C. Keruly, Andrew O. Westfall, Gary Marks

**Affiliations:** 1 Division of HIV/AIDS Prevention, Centers for Disease Control and Prevention, Atlanta, Georgia, United States of America; 2 ICF Macro International, Inc., Atlanta, GA, United States of America; 3 Psychiatry and Behavioral Sciences, University of Miami Miller School of Medicine, Miami, FL, United States of America; 4 Department of Health Policy & Management, Boston University School of Public Health, Boston, MA, United States of America; 5 Section of Infectious Diseases, Department of Medicine, Boston University School of Medicine, Boston, MA, United States of America; 6 Center for Healthcare Organization and Implementation Research, ENRM VA Hospital, Bedford, MA, United States of America; 7 Department of Medicine, Baylor College of Medicine, Houston, TX, United States of America; 8 STAR Clinic, Department of Medicine, SUNY Downstate Medical Center, Brooklyn, NY, United States of America; 9 Department of Medicine, Johns Hopkins University School of Medicine, Baltimore, MD, United States of America; 10 Department of Biostatistics, University of Alabama-Birmingham School of Public Health, Birmingham, AL, United States of America; Alberta Provincial Laboratory for Public Health/ University of Alberta, CANADA

## Abstract

HIV RNA viral load (VL) is a pivotal outcome variable in studies of HIV infected persons. We propose and investigate two frameworks for analyzing VL: (1) a single-measure VL (SMVL) per participant and (2) repeated measures of VL (RMVL) per participant. We compared these frameworks using a cohort of 720 HIV patients in care (4,679 post-enrollment VL measurements). The SMVL framework analyzes a single VL per participant, generally captured within a “window” of time. We analyzed three SMVL methods where the VL binary outcome is defined as suppressed or not suppressed. The omit-participant method uses a 8-month “window” (-6/+2 months) around month 24 to select the participant’s VL closest to month 24 and removes participants from the analysis without a VL in the “window”. The set-to-failure method expands on the omit-participant method by including participants without a VL within the “window” and analyzes them as not suppressed. The closest-VL method analyzes each participant’s VL measurement closest to month 24. We investigated two RMVL methods: (1) repeat-binary classifies each VL measurement as suppressed or not suppressed and estimates the proportion of participants suppressed at month 24, and (2) repeat-continuous analyzes VL as a continuous variable to estimate the change in VL across time, and geometric mean (GM) VL and proportion of participants virally suppressed at month 24. Results indicated the RMVL methods have more precision than the SMVL methods, as evidenced by narrower confidence intervals for estimates of proportion suppressed and risk ratios (RR) comparing demographic strata. The repeat-continuous method had the most precision and provides more information than other considered methods. We generally recommend using the RMVL framework when there are repeated VL measurements per participant because it utilizes all available VL data, provides additional information, has more statistical power, and avoids the subjectivity of defining a “window.”

## Introduction

HIV RNA viral load (VL) is a pivotal outcome variable in studies of HIV infected persons. Viral load measures are central to clinical trials of new antiretroviral (ART) therapy regimens [1, 2], randomized trials of ART adherence [3], and observational cohort studies of HIV patients [4–12]. In addition, VL is a key component of surveillance databases that provide information on the continuum of care of HIV patients [13, 14]. Hence, VL is an essential outcome variable across a wide spectrum of HIV research and surveillance studies.

There are several methods for analyzing VL as the outcome variable, and there are important differences among these methods. We conceptualize these methods using two frameworks: (1) use of a single measure VL (SMVL) per person, in which the single VL may have been selected from among multiple VLs available during a follow-up period for that person and (2) using all repeated VL measurements (RMVL) available during a follow-up period. Using the SMVL framework when there are several VL measurements available during a follow-up period necessitates choosing each participant’s VL measurement for inclusion in the analysis. Studies that analyze VL at a single time-point after enrollment often use an analysis interval (“window”) to capture that single value (e.g., 12 months after enrollment +/- 60 days). This approach requires the investigator to decide how to analyze subjects with VL values that lie outside the “window” (e.g., whether to exclude, include, or impute these subjects’ VL measurements). Limitations of this approach include ignoring within-participant variability, potential loss of information, and lower statistical power which can result in erroneous or misleading conclusions.

In contrast to the SMVL framework, the RMVL framework provides additional power, flexibility, and uses all available information. Flexibility of the RMVL framework is demonstrated by the ability to use repeated measures statistical models that may incorporate random effects for the intercept (baseline VL) and slope (VL trend across time) for each participant; something the SMVL framework cannot do because it uses only one measurement per participant. The RMVL framework provides additional information because models within this framework may estimate the geometric mean (GM) VL over time as well as estimate the VL for each participant at any specified follow-up time. In addition, one can obtain the proportion of participants who are virally suppressed at the specified follow-up time without using an arbitrarily defined “window.”

Using data from a recent retention in care (RIC) study, we investigate the SMVL and RMVL frameworks for analyzing VL data. Our purpose is to describe and compare several analytic methods for analyzing VL data under each of these frameworks, articulate their strengths and limitations, offer insights to guide selection of a model most suitable for the intent of an investigation, and compare results obtained using these methods when applied to an observational cohort study of HIV patients.

## Methods

### Modeling VL Suppression Using the Single Measure Framework (SMVL)

The VL outcome is often defined as a dichotomous variable based on VL suppression, where VL below a threshold, e.g., <200 copies/mL, is defined as suppressed [6, 7]. Viral load suppression is viewed as the desired outcome in longitudinal studies of HIV infected patients, where patients are followed over time after an initiating event (e.g., beginning ART treatment) or enrolling in an intervention and viral suppression determined at a specific time-point.

**Fig 1:**
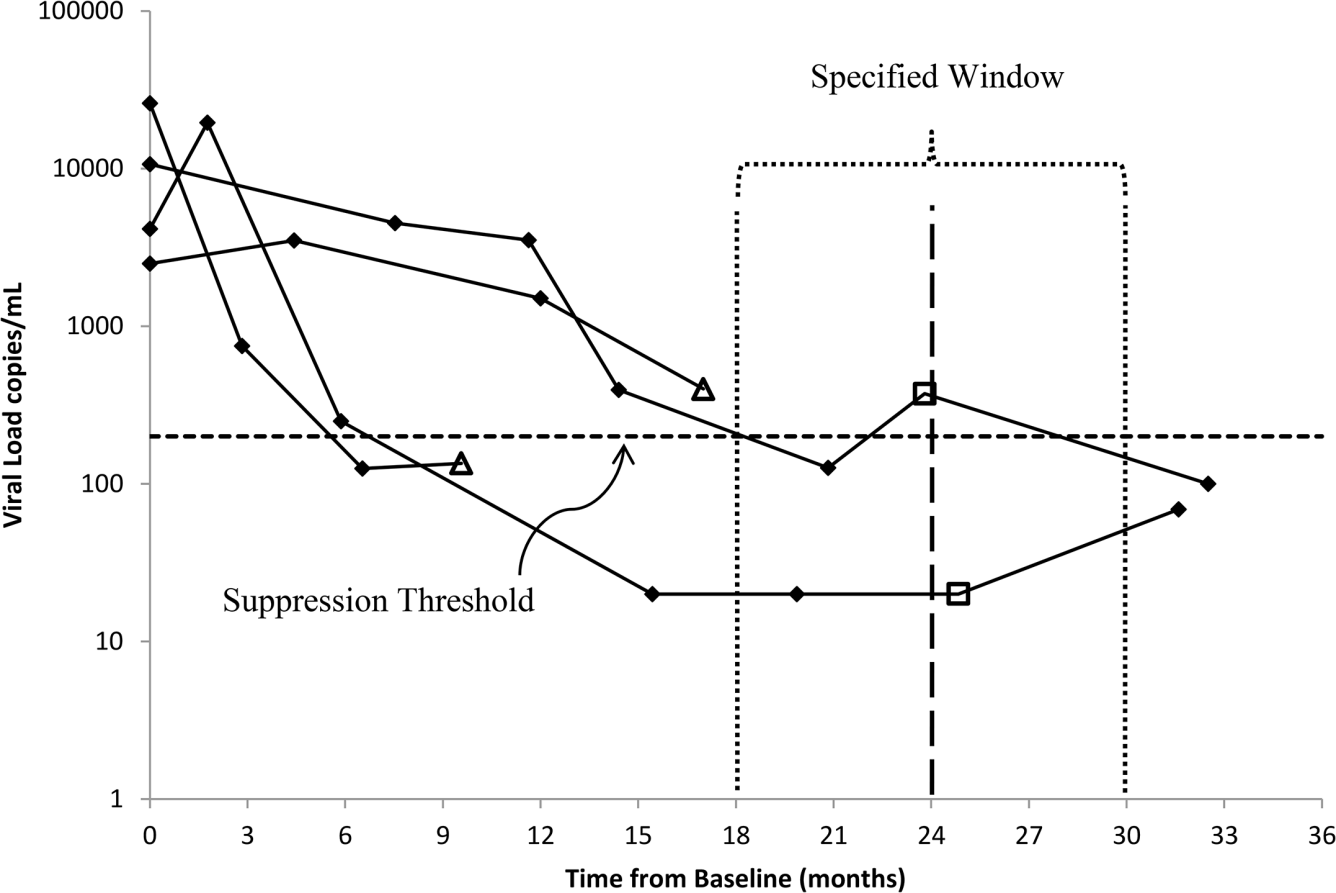
An example of log_10_(VL) copies/mL collected over time from baseline (month zero) and at subsequent follow-up visits for four participants. The triangles and squares represent the closest VL to month 24 for each of the four participants.

We investigated three commonly used analysis methods within the SMVL framework, which we illustrate using four hypothetical patients and their VL measurements (Fig 1). In all SMVL analyses we modeled VL as a dichotomous variable at a single specified follow-up time by classifying VL as suppressed or not suppressed. The first method uses only participants who have a VL measurement within the specified “window,” and if a participant had more than one VL measurement within the “window” we used the measurement closest to the specified follow-up time-point. If a participant lacked a VL within the “window” (triangles in Fig 1) then they were dropped from the analysis and we refer to this method as omit-participant. The second method, set-to-failure, used the same participants and their selected VL measurements as the omit-participant method; additionally, participants who lacked a VL within the “window” were included in the analysis and treated as non-suppressed. The third method, closest-VL, expands the concept of a “window” by using all participants and their closest VL measurements to the specified follow-up time, which implies the participant’s closest VL may be outside the “window.” In our analysis of these three methods using patient cohort data, our primary outcome is the proportion of participants who are virally suppressed at the specified time-point.

### Modeling VL using the Repeated Measures Framework (RMVL)

Our RMVL framework uses all VL measurements for all participants to estimate the geometric mean (GM) VL and/or proportion of participants who are virally suppressed at the specified follow-up time. Using our Fig 1 example, the RMVL framework uses all 18 VL measurements collected during the follow-up period as well as the four baseline measurements for the four hypothetical participants. Here we present two analysis methods using the RMVL framework.

The first RMVL method, the repeated measures binary method (repeat-binary), expands the SMVL framework for the dichotomous analysis of VL suppression at a single follow-up time by using all VL measurements available from baseline onward for each participant. For example, in Fig 1 all VL measurements below and above the threshold for viral suppression are categorized as suppressed or non-suppressed, respectively. We modeled the repeat-binary data using an appropriate model for binary data, e.g., log-binomial, and accounted for correlation within a person due to repeated measurements (e.g., generalized estimating equations) (see S1 Appendix for model specification). We included the time, calculated as VL measurement date minus the enrollment date, as a variable in the model to allow us to estimate the proportion suppressed at the specified (or any desired) follow-up time.

The second RMVL method used all available VL data from each participant and modeled log_10_(VL) as a continuous variable (repeat-continuous method). Modeling VL as a continuous variable using a random effects model allowed us to estimate individual patient’s VL, and the group GM VL, in addition to estimating the proportion suppressed. Because VL data are subject to lower and / or upper limit of detection (LOD), values that vary by the sensitivity of the assay, we accounted for LOD values using the repeat-continuous method with the Gaussian probability density and cumulative density functions (pdf-cdf) mixture distribution [15–17] (see S1 Appendix 1 for model description). The repeat-continuous method included a participant-specific random effect for the intercept (baseline VL) and slope (VL trend over time) and the method can predict each participant’s VL change over time and VL at the specified follow-up time. In addition, we calculated the group GM VL at month 24 by setting the slope and intercept random effects to zero. We summarized the participant slopes and predicted VL at the specified follow-up time using summary statistics and graphical methods, e.g., box plots. In addition, we classified each participant’s predicted VL at the specified follow-up time as suppressed or not suppressed. Lastly, to obtain a CI about the virally suppressed proportions, we used bootstrapping [18].

## Data for Modeling VL Suppression

### Study Background

The data used for the analysis came from a retention in care trial [19] that was conducted at six HIV clinics in the U.S., located in Baltimore, Birmingham, Boston, Brooklyn, Houston, Miami. Each participant provided written informed consent, and the study was approved by the Institutional Review Boards at each participating clinic site. The six clinical sites are University of Miami Miller School of Medicine, Boston University School of Medicine, Baylor College of Medicine, SUNY Downstate Medical Center, Johns Hopkins University School of Medicine, and University of Alabama-Birmingham Department of Medicine. Patients were eligible for inclusion if they had missed a primary care visit in the past 12 months, or had a gap in care ≥6 months, or were new to the clinic. The trial randomized enrollees to intervention and control arms and examined the effect of the intervention on improving attendance for primary care. The intervention significantly improved clinic attendance, but did not detect a significant effect on VL. Our analysis used VL measurements from the time of enrollment through up to 26 months of observation. Viral load measurements were obtained from patients during regularly scheduled primary care visits and not special study visits. The VL measurements obtained from patients were not equally spaced in time and the number of primary care visits varied substantially among patients.

HIV RNA VL data (copies/mL and an indicator for upper and lower LOD) as well as dates of laboratory tests were supplied by each clinic. A chart review was used to determine if participants were on ART at their enrollment (baseline) and, if not, if the participant started ART during the 12 months after baseline. Demographic variables (age and race/ethnicity) were obtained from self-reports on an audio computer-assisted self-interview (ACASI) completed by each RIC study participant at time of enrollment.

### Statistical Analysis

We omitted patients from the analysis who were virally suppressed (<200 copies/mL) at baseline because, as a group, they had no substantial variation in their baseline and post-enrollment visit(s) viral load measurements and comprised over half of the participants. Our analytic sample included patients who were not virally suppressed at baseline and had at least one post-enrollment VL measurement (720 of the 1838 patients who enrolled in the RIC trial).

Descriptive statistics were computed using the overall cohort of 720 and stratified by age group (18–29, 30–39, ≥ 40), race/ethnicity (white, black, Hispanic, other), and ART status. Participants were followed up to month 26; therefore our “window” was defined as 6 months (180 days) prior and 2 months (60 days) after month 24. We treated log_10_(VL) as a continuous variable and calculated the GM VL and 95% confidence interval (CI) using the baseline VL measurements for the 720 participants. In addition, we summarized all VL measurements after enrollment by calculating the proportion of VL measurements for all 720 participants (4679 post-enrollment visits) that were suppressed. Lastly, we computed the time from month 24 to the closest VL measurement for each participant to depict the number of participants with VLs within and outside of our defined “window” around month 24. All statistical analyses were conducted using SAS software 9.3 [20].

For each of the SMVL methods (omit-participant, set-to-failure, closest-VL) we estimated the proportion of participants (overall and by characteristic, using univariable models for race/ethnicity, age, and ART status) who were virally suppressed (VL <200 copies). In addition, we calculated the 95% CI of the proportion, risk ratio (RR) for race/ethnicity and age subgroup comparisons, and RR 95% CI using a log-binomial model with α <0.05 as significant (see S1 Appendix for model details). All three analysis methods within the SMVL framework required deciding how to treat the time-varying covariate ART when modeling at a single point in time. We modeled ART status using two methods: baseline ART (yes/no) and was ART prescribed at baseline or during the first 12 months of a patient’s observation after enrollment (baseline+12m: yes/no).

The first RMVL method (repeat-binary) modeled the binary outcome variable (suppressed / not suppressed) using all baseline and post-enrollment VL measurements to estimate the VL suppression proportions over time. We used a log-binomial model and GEE with an exchangeable covariance structure to account for repeated measurements within a participant (see S1 Appendix for model details). Each univariable model includes a stratification variable (e.g., age), continuous time (months) calculated as the post-enrollment VL date minus the baseline VL date, and stratification variable by time interaction to estimate the proportion of participants who are virally suppressed at month 24. The stratification by time interaction allows for a separate slope for each stratification subgroup. We used the repeat-binary model to estimate the proportion with viral suppression, proportion 95% CI, RR, and RR 95% CI at month 24. Our repeat-continuous RMVL method modeled log_10_(VL) as a continuous variable to estimate the GM VL and proportion of participants that are virally suppressed at month 24. Our model treated VL measurements at the lower and upper LOD as censored using the Gaussian cdf and 1–cdf, respectively. The VL measurements that are not censored are modeled using the Gaussian pdf. Each univariable model includes the stratification variable (e.g., race/ethnicity), time (months), and stratification variable by time interaction. In addition, we included a random intercept (baseline VL) and slope (VL trend over time) for each participant. The GM VL and 95% CI at month 24 for each characteristic were estimated by setting the random effects to zero. We used the estimated participant-specific intercept and slope to predict VL at month 24 for each participant and classified these predictions as suppressed or not suppressed using the threshold of <200 copies. These virally suppressed proportions from the model were summarized for the stratification variables (e.g., race/ethnicity). The 95% CI for the proportion and CI width were estimated using bootstrapping [18] (see S1 Appendix for model details).

## Results

**Fig 2:**
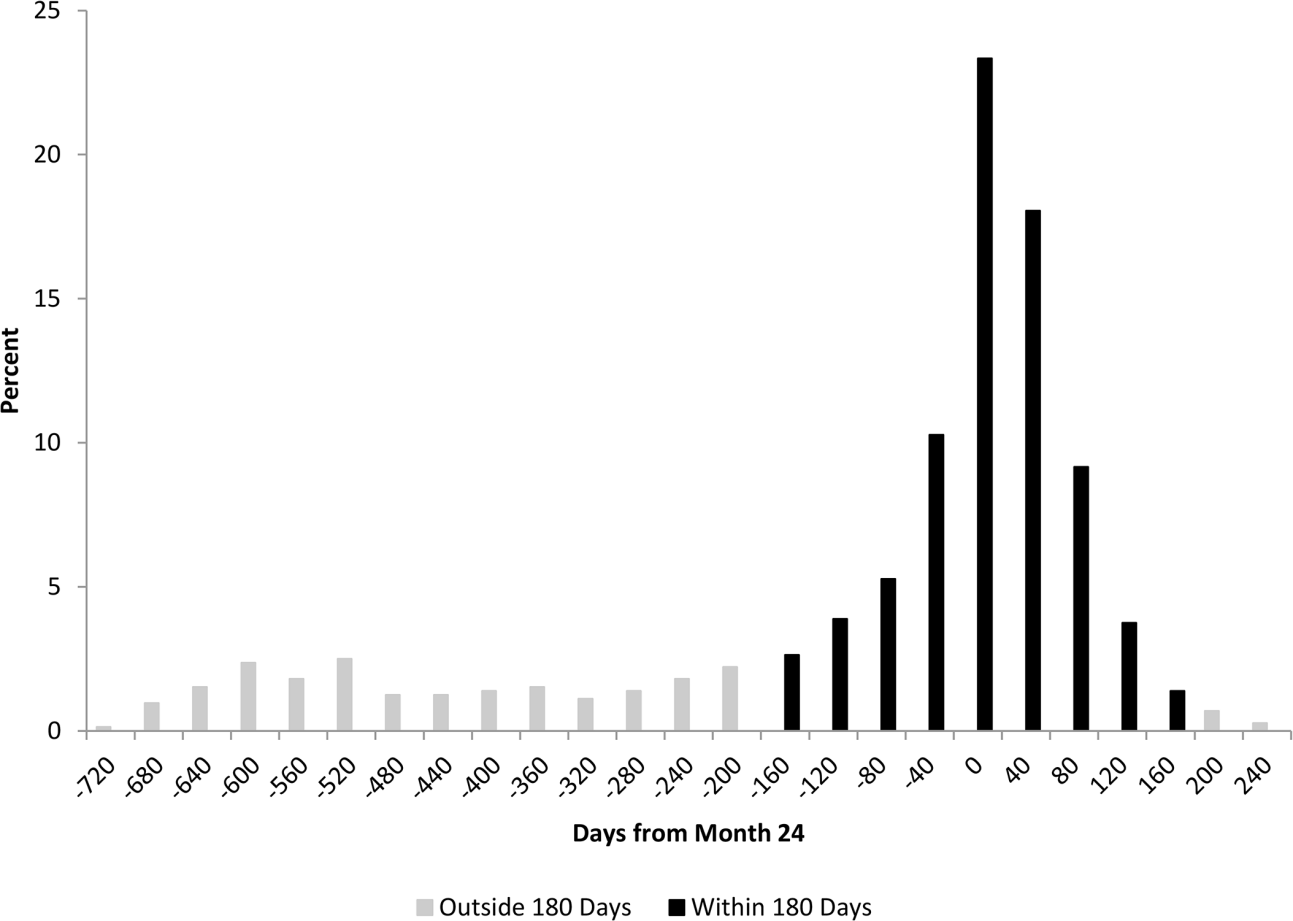
The retention in care (RIC) study distribution of each participant’s closest VL copies/mL to month 24, where zero represents month 24. There are 551 (76.5%) participants who have their closest VL within 6 months of month 24 and 232 (32.2%) whose closest VL is within 30 days.

Among the 720 participants, most were ≥40 years of age (61.3%), male (63.6%), black (73.2%), and on ART at baseline (57.5%) (Table 1). The highest GM VL copies/mL at baseline were for age 18–29 (15838), male (11627), black race (10754), and not on ART at baseline (19159). The median number of VL measurements per participant during the entire study was 6 (IQR 4, 9) with the median days between VL measurements of 90 days (IQR 55, 130). Of the 4679 post-enrollment VL measurements, 2443 of them were <200 (52.2%), and the percent of post-enrollment VLs <200 was highest for age 18–29 (53.9%), male (54.7%), white (64.2%), and on ART at baseline (55.2%). The time from month 24 to the participant’s closest VL reveals a total of 551 participants (76.5%) had their closest VL within 6 months (180 days) before or 2 months (60 days) after month 24, including 232 (32.2%) within 30 days (Fig 2). Of the 169 participants (23.5% of 720) with their closest VL before 6 months prior to month 24, there were 106 (14.7% of 720) >360 days from month 24. Of the 106 participants with their closest VL >360 days from month 24 there were 53 (7.4%) who had their closest VL to month 24 within 6 months of baseline. The median time from month 24 to the participant’s closest VL was 28 days prior to month 24 (IQR -144, 14).

**Table 1:** The baseline calculated geometric mean (GM) viral load (VL), GM VL 95% confidence interval (CI), and proportion of all visits for all participants with VL <200 (suppressed) after baseline for each characteristic.

Characteristic	Baseline VL copies/mL (N = 720)			Proportion of Records with VL <200 after Baseline (N = 4679)		
	N	GM VL	GM VL 95% CI	VL <200 (n = 2443)	VL ≥200 (n = 2236)	Percent
**Overall**	720	9975	8436, 11795	2443	2236	52.2
**Age (years)**						
18–29	106	15838	10262, 24443	375	321	53.9
30–39	172	12748	9065, 17927	528	522	50.3
40+	441	8101	6548, 10021	1540	1385	52.6
Missing	1			0	8	0.0
**Sex**						
Male	458	11627	9436, 14327	1594	1318	54.7
Female	261	7490	5681, 9876	849	906	48.4
Transgender	1			0	12	0.0
**Race / Ethnic**						
White	80	9456	5723, 15624	300	167	64.2
Black	527	10754	8844, 13076	1727	1763	49.5
Other	21	7394	2777, 19686	77	57	57.5
Hispanic	92	7273	4555, 11612	339	249	57.7
**ART Baseline**						
Yes	414	6157	4970, 7628	2308	1873	55.2
No	306	19159	14933, 24581	135	363	27.1

Analysis results for modeling VL suppression using the SMVL framework (omit-participant, set-to-failure, closest-VL) and RMVL framework (repeat-binary, repeat-continuous) are presented in Tables 2 and 3, respectively. Under the SMVL framework, the omit-participant method used 551 (76.5%) of the 720 participants who had a VL measurement within the “window” around month 24; whereas set-to-failure and closest-VL methods used all 720 participants (Fig 2). Under the RMVL framework, both the repeat-binary and the repeat-continuous used the 720 participants’ baseline VL measurements and all 4679 VL post-enrollment measurements. There was substantial variability among the methods using the SMVL framework for the estimated percent of participants who were virally suppressed at month 24. The estimated percentage and associated 95% CI for VL suppression at month 24 were omit-participant 58.8 (54.8, 63.1), set-to-failure 45.0 (41.5, 48.8), and closest-VL 54.2 (50.7, 57.9). The repeat-binary and repeat-continuous methods using the RMVL framework had similar VL suppression percent estimates of 58.5 (55.2, 61.9) and 56.7 (55.3, 59.2), respectively. Moreover, the 95% CI width was substantially smaller using the RMVL framework repeat-continuous method.

**Table 2:** The estimated number and percentage of participants virally suppressed (VL <200) at month 24 using the omit-participant, set-to-failure, and closest-VL analysis performed within the SMVL framework using data from the RIC study.

Characteristic	Omit-participant (N = 551)				Set-to-Failure (N = 720)				Closest-VL (N = 720)			
	Number VL <200	Percent (95% CI)	Risk Ratio (RR)	RR 95% CI	Number VL <200	Percent (95% CI)	Risk Ratio (RR)	RR 95% CI	Number VL <200	Percent (95% CI)	Risk Ratio (RR)	RR 95% CI
**Overall**	324	58.8 (54.8, 63.1)	NA	NA	324	45.0 (41.5, 48.8)	NA	NA	390	54.2 (50.7, 57.9)	NA	NA
**Age (years)**												
18–29	35	47.9 (36.8, 59.3)	REF		35	33.0 (24.8, 42.5)	REF		46	43.4 (34.3, 53.0)	REF	
30–39	73	57.9 (49.2, 66.2)	1.21	0.91, 1.60	73	42.4 (35.3, 49.9)	1.29	0.93, 1.77	91	52.9 (45.4, 60.3)	1.22	0.94, 1.58
40+	216	61.5 (56.3, 66.5)	1.28	0.997, 1.65	216	49.0 (44.3, 53.6)	1.48	1.11, 1.98	253	57.4 (52.7, 61.9)	1.32	1.05, 1.67
**Race / Ethnic**												
White	41	70.7 (57.8, 80.9)	REF		41	51.3 (40.4, 62.0)	REF		51	63.8 (52.7, 73.5)	REF	
Black	226	55.0 (50.2,59.7)	0.78	0.65, 0.94	226	42.9 (38.7, 47.2)	0.84	0.66, 1.06	270	51.2 (47.0, 55.5)	0.80	0.67, 0.97
Other	10	76.9 (47.9, 92.4)	0.96	0.76, 1.21	10	47.6 (27.9, 68.2)	0.93	0.57, 1.53	12	57.1 (36.0, 76.0)	0.90	0.60, 1.34
Hispanic	47	68.1 (56.3, 78.0)	1.09	0.77, 1.53	47	51.1 (41.0, 61.1)	1.00	0.74, 1.34	57	62.0 (51.7, 71.3)	0.97	0.77, 1.22
**ART Baseline**												
Yes	181	57.1 (51.6, 62.4)	0.93	0.81, 1.07	181	43.7 (39.0, 48.5)	0.94	0.80, 1.10	216	52.2 (47.4, 57.0)	0.92	0.80, 1.05
No	143	61.1 (54.7, 67.2)	REF		143	46.7 (41.2, 52.3)	REF		174	56.9 (51.3, 62.3)	REF	
**ART Baseline + 12 months**												
Yes	294	60.5 (56.1, 64.8)	1.31	0.998, 1.72	294	46.2 (42.4, 50.1)	1.29	0.96, 1.75	357	56.1 (52.3, 59.9)	1.43	1.09, 1.88
No	30	46.2 (34.5, 58.3)	REF		30	35.7 (26.2, 46.5)	REF		33	39.3 (29.5. 50.1)	REF	

The ART baseline and ART baseline +12 months are defined as the participant being on ART at the start of the study and either at the start of study or the first 12 months of the study, respectively.

Notes: The omit-participant method removes participants from the analysis who do not have a VL within the 12-month “window” (-6/+2 months) around month 24. The set-to-failure method sets all VL for those participants without a VL measurement within the “window” to non-suppressed (failure). Lastly, the closest-VL method uses the closest VL measurement for each participant to month 24.

**Table 3:** The estimated percent of participants virally suppressed (VL <200) at month 24 and risk ratio (RR) using the RMVL framework analysis methods repeat-binary and repeat-continuous using data from the retention in care (RIC) study.

Characteristic	Repeat-Binary				Repeat-Continuous							
	Percent <200 at Month 24	Percent 95% CI	Risk Ratio (RR)	RR 95% CI	GM VL	GM VL 95% CI	GM VL Ratio	GM Ratio 95% CI	Percent <200 at Month 24	Percent 95% CI	Risk Ratio (RR)	RR 95% CI
**Overall**	58.5	55.2, 61.9	NA	NA	115	81, 162	NA	NA	56.7	55.3, 59.2	NA	NA
**Age (years)**												
18–29	57.4	49.0, 67.1	REF		134	54, 334	REF		54.7	50.9, 58.5	REF	
30–39	57.3	50.4, 65.1	0.999	0.82, 1.22	110	54, 223	0.82	0.26, 2.59	55.8	53.5, 61.1	1.02	0.96, 1.14
40+	59.3	55.3, 63.6	1.03	0.87, 1.23	110	71, 170	0.82	0.30, 2.24	57.8	55.8, 60.1	1.06	0.97, 1.14
**Race / Ethnic**												
White	79.3	70.1, 89.6	REF		13	4, 37	REF		77.5	71.3, 80.0	REF	
Black	57.9	54.0, 62.2	0.73	0.63, 0.84	171	116, 253	13.32	4.29, 41.39	51.4	50.3, 54.7	0.66	0.64, 0.75
Other	71.5	57.6, 88.7	0.90	0.70, 1.16	62	8, 450	4.80	0.50, 45.80	71.4	61.9, 76.2	0.92	0.80, 1.05
Hispanic	68.1	58.9, 78.7	0.86	0.71, 1.04	78	30, 200	6.07	1.47, 25.13	69.6	60.9, 71.7	0.90	0.80, 0.98
**ART**												
Yes	64.7	61.1, 68.5	1.67	1.27, 2.18	109	78, 154	0.09	0.04, 0.24	57.2	57.0, 60.4	1.72	1.46, 1.95
No	38.9	29.8, 50.7	REF		1181	480, 2905	REF		33.3	29.9, 40.2	REF	

The group geometric mean (GM) VL, GM VL ratio, and GM VL ratio 95% CI was estimated at month 24 using the repeat-continuous method. The ART variable is a time-varying covariate.

Results for the methods using the SMVL and RMVL frameworks illustrate that the repeat-continuous method provided a substantial gain in power compared to all other considered methods (S1 and S2 Tables). We compared the VL suppression proportion’s 95% CI width for the omit-participant, set-to-failure, closest-VL, and repeat-binary methods to the repeat-continuous method as the reference. The overall proportion suppressed CI width ratio ranged from 1.72 (binary-repeat) to 2.12 (omit-participant). For the age and race/ethnicity subgroups, the CI width ratio ranged from 1.81 (closest-VL; Hispanic) to 3.11 (omit-participant; other race). In general, the proportion suppressed CI width ratios for the omit-participant, set-to-failure, closest-VL, and repeat-binary were similar to each other and the proportion suppressed CI width was substantially wider than the repeat-continuous CI width. The repeat-binary had the smallest CI width ratio compared to repeat-continuous method except for the comparison of other race versus white, where it is 1.84 and the omit-participant is 1.80, which indicates the repeat-binary generally improves the precision compared to the SMVL methods. The CI width ratio ranged from 1.80 (omit-participant; other race versus white) to 5.12 (set-to-failure; 40+ versus 18–29). Set-to-failure had the highest CI width ratio (range: 3.64–5.12) in all comparisons except for Hispanic versus white, where the omit-participant method had the highest CI width ratio (4.22).

**Fig 3:**
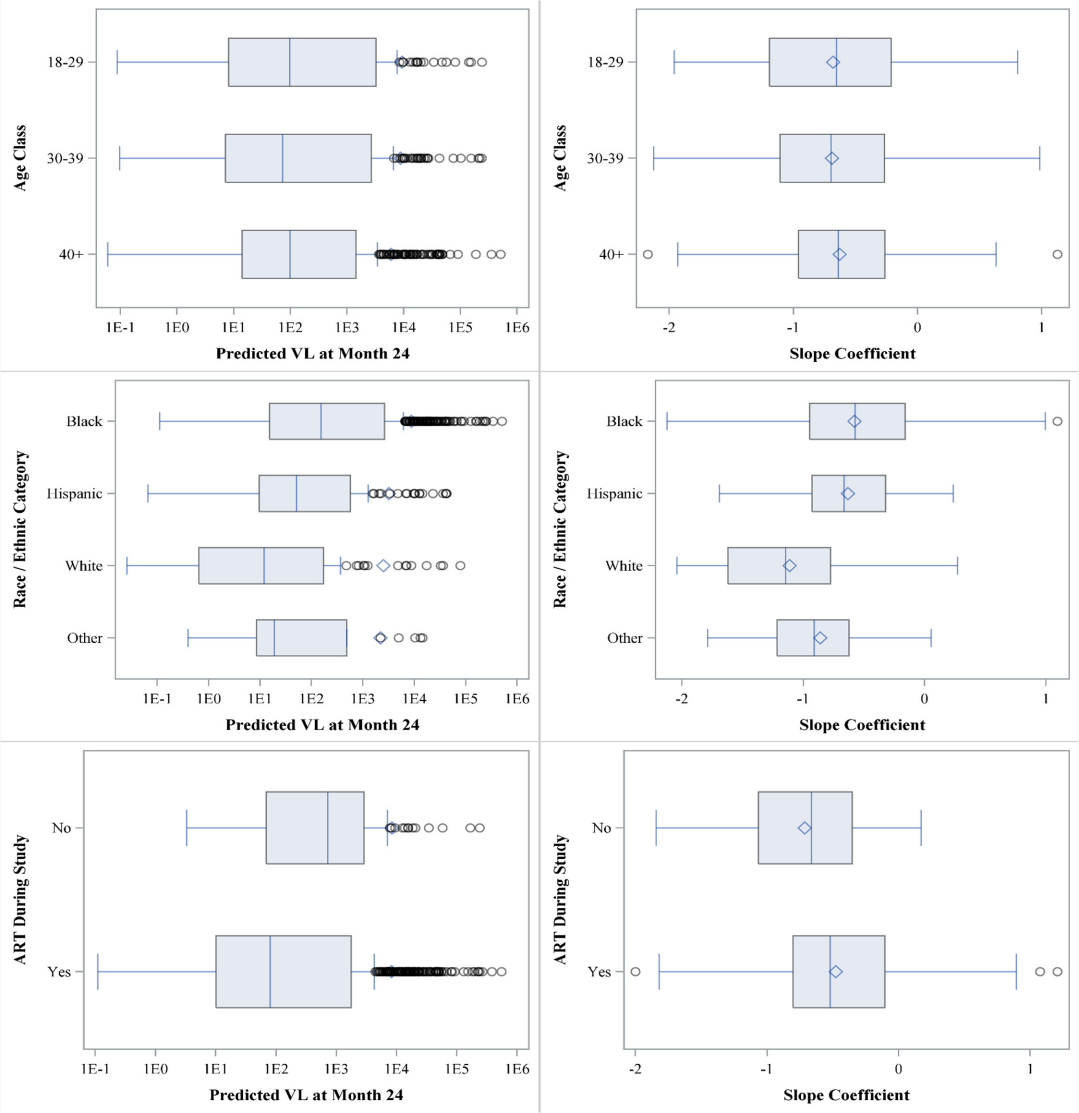
Box plots by characteristic for the participants predicted VLs at month 24 and rate of change (slope). The shaded box represents the 25^th^ and 75^th^ percentiles, while the vertical line and diamond within the shaded box are the median and mean, respectively. The upper and lower arms, represented by vertical lines, are the 2.5 and 97.5 percentiles, and dots outside these arms are considered outliers.

**Fig 4:**
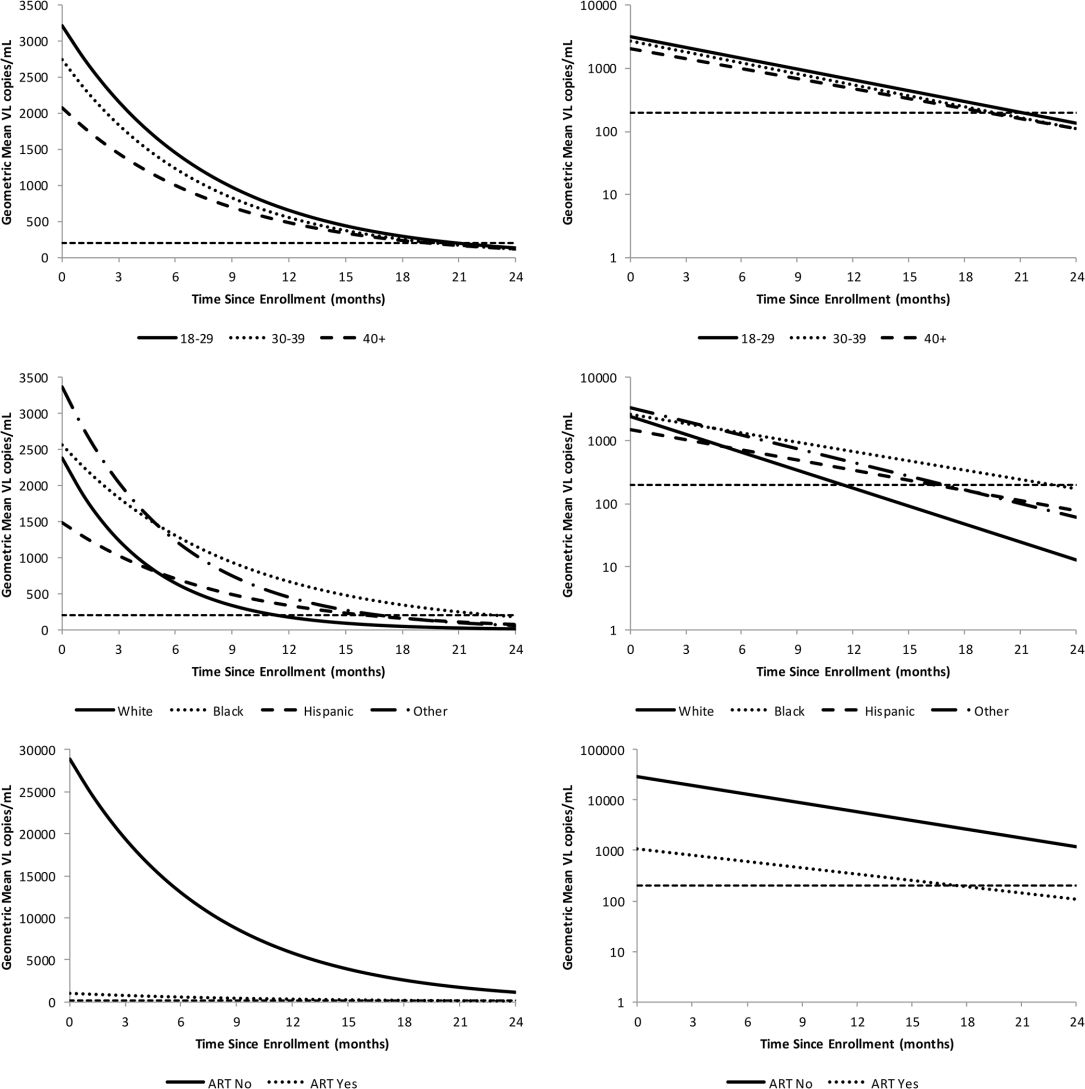
The predicted geometric mean (GM) by characteristic using the pdf-cdf random effects model plotted on the VL and log_10_(VL) scales. The horizontal line is the defined level of suppression (<200 copies/mL).

The repeat-continuous method was the only method considered that can estimate the VL, both the individual and group GM, and results revealed that the lowest estimated GM VL at month 24 for age, race/ethnicity, and ART are age classes 30–39 and 40+ (110, 95% CI of 54–223 and 71–170, respectively), whites (13, 95% CI: 4, 37), and on ART (109, 95% CI 78–154) (Table 3). In addition, the ART “no” is the only considered characteristic with an estimated GM VL at month 24 >200 copies (1181; 95% CI: 480, 2905). We summarized the predicted participant-specific slopes and viral suppression at month 24 using box plots and descriptive statistics (Fig 3). Box plots revealed that white race (compared to black and Hispanic) and ART baseline+12m (compared to no ART at baseline or during the first 12 months) had substantially lower estimated median GM VL at month 24. Those participants not on ART at baseline or within 12 months (compared to using ART during first 12 months) and black race (compared to the other race/ethnicity groups) had the highest percent of participants with VL >200 at month 24, 66.7% and 48.6%, respectively. Box plots illustrated the percent of participants by characteristic that had a slope >0 (positive slope indicates their VL is increasing over time). The factors with the greatest percentage of participants with a slope >0 were black (15.0) and age 18–29 (38.7) with white (2.5) and not on ART (3.6) having the smallest percentage of participants with a positive slope. White race and not being on ART had the steepest, i.e., most negative, median slope, which means they had the greatest rate of change in GM VL from baseline to month 24. Lastly, we summarized the repeat-continuous model results for the GM VL change over time by characteristic (Fig 4). There was substantial variability among the participant’s estimated GM VL slopes for race/ethnicity. Furthermore, the participants who were never on ART failed to have their estimated GM VL reach the threshold (VL <200) by month 24. The estimated time to the threshold (VL < 200) for the GM VL was shortest for race white (11.6 months), Hispanic (16.3 months), and “other” race (16.9 months), whereas the longest times to threshold, in months, were for patients not on ART (37.3) and black (22.6).

## Discussion

We investigated two general frameworks for analyzing VL data. One was a repeated measures viral load (RMVL) framework, and the other was a single measure viral load (SMVL) framework. Our investigation demonstrated the RMVL framework performed exceptionally well using data from a RIC trial that enrolled participants who had not been attending clinic regularly. Hence, the RMVL framework performed exceptionally well for participants who likely have more sporadic VL data, i.e., few VL measurements over time compared to participants who are currently engaged in care, which further demonstrates the value of the RMVL framework. The RMVL framework has an advantage over the SMVL framework because it utilizes all available baseline and follow-up VL information for all participants. Moreover, in contrast to the three SMVL methods and the RMVL repeat-binary method, which can only estimate the proportion of participants that are virally suppressed, the repeat-continuous method of the RMVL framework can also estimate the VL for each participant as well as the GM VL. Furthermore, the repeat-continuous method allows us to obtain the estimated VL change over time (slope) for each participant in addition to the GM VL slope. The RMVL framework is flexible and nullifies the necessity of specifying an arbitrary “window” to estimate the GM VL and / or VL suppression at the specified follow-up time, which eliminates the need to define the status of participants lacking a VL measurement within the “window.” Furthermore, the RMVL framework can provide an estimate of the GM VL or proportion virally suppressed throughout the range of the follow-up time period.

Methods that analyze VL suppression within the SMVL framework have few advantages but several disadvantages. The primary advantage of the SMVL framework using a dichotomous outcome variable is the simplicity of the analysis using any software package. There are several disadvantages of the SMVL framework compared to the RMVL framework. First, when using the SMVL framework and analyzing a single follow-up VL measurement with a suppression threshold we are grouping participants marginally >200 copies/mL with participants who may have large values for VL, which may be clinically questionable. Second, a loss of power generally results when discarding repeated VL measurements and analyzing only one VL measurement per participant [21]. Even if all participants have only one follow-up VL measurement within the “window” or exactly at the ending follow-up time there would likely be a loss of power due to dichotomizing a continuous variable [22, 23]. Third, investigators have to determine how to analyze, if at all, VL measurements outside the “window.” All methods analyzing VL as a dichotomous suppressed or not suppressed outcome are limited to only estimating the proportion suppressed. The repeat-binary, unlike the SMVL methods, can estimate the proportion suppressed at any point over the range of the follow-up times and estimates the rate of change in viral suppression. Two additional methods recently used within the SMVL framework include time to VL suppression and viremia copy-years (VCY) [24]. However, both of these methods either collapse multiple VL measurements per participant into a single VL measure (VCY) or ignore the repeated VL measurements by modeling the time to first VL suppression.

All considered methods within the SMVL framework, whether using a “window” or not, assume that the participant’s observed VL is a reasonable prediction of the participant’s VL at the desired follow-up time. Although not normally stated, the participant’s VL within the “window” or the closest-VL is a prediction for the specified follow-up time-point. The omit-participant method may appeal to analysts because of its simplicity, but the method discards information on participants who lack a VL measurement within the “window.” Hence, the omit-participant method discards participants from the analysis who may have ≥1 follow-up VL measurements (Fig 1) but lack a VL within the “window,” which may result in an additional loss of power due to a decrease in the sample size. In addition, the omit-participant analysis ignores potential differences between participants with and without a VL within the “window,” and the resulting inference based only on participants with a VL within the “window” may not be applicable to the entire participant population. This problem is especially important for observational studies assessing viral suppression where participants being lost over time may be informative. The set-to-failure method assumes all participants lacking a VL within the specified “window” are VL suppression failures, which is likely to misclassify some participants. For example, in our data analysis there are 169 (23.5%) of the 720 participants without a VL within the “window” (Fig 2). However, of these 169 participants 66 (39.1%) were virally suppressed at their last follow-up VL measurement, which suggests that some of the 169 participants would be virally suppressed if they had a VL measurement within the “window.” In addition, the set-to-failure method may substantially reduce the estimated proportion suppressed as all failure participants would contribute to the denominator but the number of successful suppressions would remain unchanged from the omit-participant method. Moreover, the set-to-failure method for our data tends to have the largest estimated SE, making the method less likely to detect significant differences. The closest-VL method has a disadvantage in that some of the VL measurements may be far from the desired follow-up time, e.g., in our data the closest VL for 105 of 169 (62.1%) participants was greater than one year prior to the desired month 24 post-enrollment follow-up. This leads to questionable validity about using these VL measurements as predictions at month 24. Regardless of analytical method, only having VL measurements far from the specified follow-up time for some participants may be a concern.

The analytical method(s) selected by an investigator should be based on study question(s) and available, or to be collected, data. Our results illustrate there can be substantial differences based on the selected HIV VL analytical method for the estimated percent suppressed, RR, and associated 95% CIs among the three SMVL methods, with more consistent results between the two RMVL methods (Tables 2 and 3). Differences in the SMVL methods results will increase as the proportion of patients with a VL measurement outside the “window” increases. As an example, results revealed substantial differences among the methods for the age 40+ compared to 18–29 RR as the set-to-failure and closest-VL are significant, borderline significance for omit-participant, and far from significant for the repeat-binary and repeat-continuous RMVL methods (p>0.50). Given these significant results for the age 40+ compared to 18–29 RR it may be tempting to select one or all of the SMVL methods but it is inappropriate to choose an analysis method based on which analysis provides significant findings and the analysis method should be carefully determined *a priori*.

Use of the SMVL and RMVL frameworks may result in substantial differences for the estimated proportion suppressed (Tables 2 and 3). For example, the proportion of participants ages 18–29 with suppressed VL for the omit-participant (47.9), set-to-failure (33.0), and closest-VL (43.4) compared to repeat-binary (57.4) and repeat-continuous (54.7) are substantially different. In addition, the data selected for analysis, i.e., suppressed or not suppressed, using the SMVL method can be dramatically altered by changing the “window” interval and / or follow-up time. Results reveal there are 12 participants in the age class 18–29 who are classified as suppressed using the repeat-continuous method but are classified as not suppressed using the SMVL methods (S1 Fig). Data for these 12 participants reveal the pitfalls of the SMVL framework due to choosing a single VL measurement per person to summarize VL suppression. For example, if the specified post-enrollment follow-up time were changed to month 18 from month 24, then five of these 12 participants would have their VL classified as suppressed.

In summary, the RMVL framework eliminates the need to define a “window” and is a natural modeling framework for characterizing VL measurements over time and predicting the proportion of virally suppressed participants at a specified follow-up time. The RMVL framework is flexible, can accommodate a wide variety of models within the frequentist and Bayesian paradigms, and generally has greater statistical power than models within the SMVL framework. We generally recommend the RMVL framework if there are multiple VL measurements per participant and not the SMVL framework because of a loss of power, discarding of information, subjectivity in defining a “window,” and investigators having to decide how to handle participants with VL measurements outside the “window.”

## Supporting Information

S1 Appendix Details for all models and SAS code. The statistical details for all models using the single measure and repeated measures viral load (VL) frameworks. The SAS code for fitting the repeat-continuous model subject to limit of detection VL values.
(DOCX)

S1 Fig Viral load measurements over time. The 12 aged 18–29 participants for the three SMVL framework analyses (omit-participant, set-to-failure, closest-VL) that are analyzed as non-suppressed or set to missing and are predicted as suppressed using the RMVL repeat-continuous model at month 24. The triangles represent values used for the SMVL analyses and the “window” is from 540–780 days. Triangles not within the “window” are set to missing (omit-participant) or failure (set-to-failure) or non-suppressed (closest-VL method).
(TIF)

S1 Table Viral suppression among study participants. The percent of the RIC study participants with virologic suppression, percent 95% CI, 95% CI width, and CI width ratio at month 24 using the SMVL (omit-participant, set-to-failure, closest-VL) and RMVL (repeat-binary, repeat-continuous) frameworks.
(DOCX)

S2 Table Viral suppression risk ratios by method. The estimated viral suppression risk ratio (RR), RR 95% CI, RR 95% CI width, and CI width ratio at month 24 using the SMVL (omit-participant, set-to-failure, closest-VL) and RMVL (repeat-binary, repeat-continuous) frameworks.
(DOCX)
