# Supplementary material for: A Comparison of Methods for Analyzing Viral Load Data in Studies of HIV Patients
Source: PLoS One. 2015 Jun 19;10(6):e0130090. doi: 10.1371/journal.pone.0130090 (PMC4474923; doi:10.1371/journal.pone.0130090)
Supplement: S1 Appendix — The statistical details for all models using the single measure and repeated measures viral load (VL) frameworks. The SAS code for fitting the repeat-continuous model subject to limit of detection VL values. (DOCX) [file pone.0130090.s001.docx]

**Supplemental Material**

**Model Details**

All analysis methods used explanatory variables age (18-29, 30-39, 40+) and race/ethnicity (black, white, Hispanic, other). In addition, the SMVL framework methods (omit-participant, set-to-failure, and closest-VL) used two definitions of ART (yes, no): ART at baseline (ART_B_) and ART at baseline or during the first 12 months of a participant’s observation period after baseline (ART_A_). In contrast, the RMVL framework methods (repeat-binary, repeat-continuous) use ART as a time-varying covariate, which can be updated from ART no to ART yes. Each predictor variable has *p* categories and *p* – 1 parameter(s) in the models specified below.

**Modeling VL Suppression Using a Single Measure Framework (SMVL)**

We modeled VL suppression using a log-binomial generalized linear model (GLM), which is defined as a binomial distribution with a log link function, in SAS PROC GENMOD 9.3 The log-binomial model is defined by letting $y_{ij}$ denote the outcome VL suppression status [suppressed (1), not suppressed (0)] for the *i^th^* subject and *j^th^* SMVL framework method (omit-participant, set-to-failure, closest-VL). Furthermore, the explanatory indicator variables for age, race/ethnicity, ART_B,_ and ART_A_, which were modeled separately in univariable models, are given by ***X****_i_* and *β_0_* is the overall mean. The log-binomial model relates the log link, i.e., log of the risk, to the explanatory variables using:

$$\log\left(\mu_{ij}\right)=\beta_{0j}+\beta_{1j}X_{1j}+...+\beta_{p-1j}X_{p-1j}$$

**Modeling VL using the Repeated Measures Framework (RMVL)**

**Repeat-Binary Method**

We expanded the SMVL framework by using the RMVL framework for the repeat-binary and defining the binary outcome for VL suppression using a repeated measures log-binomial model. Our repeat-binary model is defined by letting $y_{ij}$ denote the outcome VL suppression status for the *i^th^* subject and *j^th^* visit. Furthermore, the indicator explanatory variables for age, race/ethnicity, and ART (time varying), which were modeled separately using univariable models, are given by ***X****_i_* and *β_0_* is the overall mean. In addition, each model includes time, which was defined as the time from baseline to the visit, and the interaction of time and the explanatory variable. The repeat-binary log-binomial models the log of the risk using the explanatory variable and time (*t*) as:

$$\log\left(\mu_{ij}\right)=\beta_{0j}+\beta_{t}t+\beta_{1j}X_{1ij}+...+\beta_{p-1j}X_{p-1ij}+\beta_{t,1}tX_{1ij}+...$$

Our repeat-binary model accounts for the correlation within a participant due to repeat VL observations using generalized-estimating-equation (GEE) with an exchangeable covariance structure. All repeat-binary models are fit using SAS PROC GENMOD 9.3.

**Repeat Continuous Method**

We used the repeat-continuous method to model the log_10_(VL) data that are subject to a limit of detection (LOD) using a mixture distribution of the Gaussian probability density function (pdf) and cumulative distribution function (cdf), which we refer to as the pdf-cdf model [15-17, 22]. We defined our pdf-cdf model by letting *d_L_* and *d_U_* denote the lower and upper LOD, respectively. Now define the indicator variable for the observed *j^th^* baseline / follow-up time VL for the *i^th^* participant as:

$$\gamma_{ij}=\left\{ \begin{aligned} 0, if y_{ij}\text{>}\text{ }d_{L}\text{ } \\ 1, if y_{ij}\text{ }\text{≤}\text{ }d_{L} \end{aligned} \right.$$

$$\delta_{ij}=\left\{ \begin{aligned} 0, if y_{ij}\text{≤}d_{L} \\ 0, if y_{ij}\text{ }\text{≥}d_{U}\text{ } \\ 1, if d_{L}<y_{ij}\text{<}d_{U} \end{aligned} \right.$$

$$\tau_{ij}=\left\{ \begin{aligned} 0, if y_{ij}\text{ }\text{<}d_{U}\text{ } \\ 1, if y_{ij}\text{ }\text{≥}d_{U} \end{aligned} \right.$$

We defined a generalized pdf-cdf model using the indicator variables $\gamma_{ij}, \delta_{ij,}, \mathrm{and} \tau_{ij}$to account for lower and upper LOD censoring as:

$$f_{ij}\left( y \right)=\left[ G_{ij}\left( y \right) \right]^{\gamma_{ij}}\left[ g_{ij}\left( y \right) \right]^{\delta_{ij}}\left[ {1-G}_{ij}\left( y \right) \right]^{\tau_{ij}}$$

where $G_{ij}\left( \cdot\right) \mathrm{and}g_{ij}\left( \cdot\right)$ are the cdf and pdf, respectively. Given the general pdf-cdf model form we defined our Gaussian pdf-cdf model as:

$$g\left( y_{ij}|\boldsymbol{\beta},\sigma\right)=\frac{1}{\left( 2\pi\sigma^{2} \right)^{\frac{1}{2}}}exp\left[ -\frac{1}{2\sigma^{2}}\left( y_{ij}-\mu_{ij} \right)^{2} \right]$$

$$G\left( d_{L}|\boldsymbol{\beta},\sigma\right)=\int_{-\infty}^{d_{L}} g\left( t|\boldsymbol{\beta},\sigma\right)dt$$

$$G\left( d_{U}|\boldsymbol{\beta},\sigma\right)=\int_{d_{U}}^{\infty} g\left( t|\boldsymbol{\beta},\sigma\right)dt$$

The mean, *µ_ij_*, is a linear function of the predictor variable(s) and defined as for the repeat-binary method (see above), and ***b*** is the subject-specific random effects parameters for the intercept and slope and ***z*** the corresponding design matrix. Hence, our model is defined as:

$$\mu_{ij}=\boldsymbol{X}_{ij}\boldsymbol{\beta+}\boldsymbol{z}_{ij}\boldsymbol{b}$$

The likelihood function for all participants and visits is given by:

$$L=\prod_{i=1}^{S} \prod_{j=1}^{N} \left[ G\left( d_{L}|\boldsymbol{\beta},\sigma\right) \right]^{\gamma_{ij}}\left[ g\left( y_{ij}|\boldsymbol{\beta},\sigma\right) \right]^{\delta_{ij}}\left[ G\left( d_{U}|\boldsymbol{\beta},\sigma\right) \right]^{\tau_{ij}}$$

Our repeat-continuous pdf-cdf model is fit by coding the likelihood using SAS PROC NLMIXED 9.3 and using the participant as the random effect for the slope and intercept (code provided below). Once we fit the model we used the estimated participant’s slope, i.e., VL change over time, to predict each participant’s VL at the specified follow-up time. In addition, we set the slope and intercept random effects to zero, the expected value on the log-scale, and then back-transformed to obtain the group GM VL.

**Repeat-Continuous Model SAS Code**

**proc** **nlmixed** data=”dataset”;

/* Provide the starting values for parameters, sigsqe is the error variance for a linear model and var_u1 / var_u2 are the variances of the random subject effect,

We could allow for the covariance to be non-zero. */

parms a0=**3.75** t0=-**0.43** sigsqe=**1.24** var_u1=**0.63** var_u2=**0.50**;

/* Calculate pi, 3.1416...... */

pi=**2***arsin(**1**);

/* Define the linear model with a random intercept (u1) and slope (u2) */

mu = a0 + t0*year + u1 + u2*year;

/* Define likelihood function for observations censored and not censored */

/* censor = 0 is just the PDF of the normal distribution for non-LOD values */

/* censor = 1 is for lower LOD censored values */

/* censor = 2 is for upper LOD censored values */

/* Note that mu is the predicted mean and lnvl (log base 10 VL) is your observed

data */

if censor=**0** then L=(**1**/(sqrt(**2***pi*sigsqe)))*exp(-(lnvl-mu)****2**/(**2***sigsqe));

if censor=**1** then L=probnorm((lnvl-mu)/sqrt(sigsqe));

if censor=**2** then L=**1** - probnorm((lnvl-mu)/sqrt(sigsqe));

/* Define the log(likelihood), model, and random effects */

LL=log(L);

model lnvl ~ general(LL);

random u1 u2 ~ N([**0**, **0**], [var_u1, **0**, var_u2]) subject=ID out=new_ran;

predict a0 + t0***2** + u1 + u2***2** out=new_u ;

estimate 'Overall GM at 24 Months' a0 + t0***2**;

ods output additionalestimates=new_pt;

**run**;

**Supplemental Table 1.** The percent of the RIC study participants with virologic suppression, percent 95% CI, 95% CI width, and CI width ratio at month 24 using the SMVL (omit-participant, set-to-failure, closest-VL) and RMVL (repeat-binary, repeat-continuous) frameworks.

| **Characteristic** | **Percent Suppressed (VL <200)** | **Percent 95% CI** | **Percent 95% CI Width** | **CI Width Ratio** |
| --- | --- | --- | --- | --- |
| **Overall** |  |  |  |  |
| Omit-participant | 58.8 | 54.8, 63.1 | 8.23 | 2.12 |
| Set-to-Failure | 45.0 | 41.5, 48.8 | 7.27 | 1.87 |
| Closest-VL | 54.2 | 50.7, 57.9 | 7.28 | 1.87 |
| Repeat-Binary | 58.5 | 55.2, 61.9 | 6.69 | 1.72 |
| Repeat-Continuous | 56.7 | 55.3, 59.2 | 3.89 | REF |
| **Age** |  |  |  |  |
| ***18 - 29*** |  |  |  |  |
| Omit-participant | 47.9 | 36.8, 59.3 | 22.5 | 2.96 |
| Set-to-Failure | 33.0 | 24.8, 42.5 | 17.7 | 2.33 |
| Closest-VL | 43.4 | 34.3, 53.0 | 18.7 | 2.46 |
| Repeat-Binary | 57.4 | 49.0, 67.1 | 18.1 | 2.38 |
| Repeat-Continuous | 54.7 | 50.9, 58.5 | 7.6 | REF |
| ***30-39*** |  |  |  |  |
| Omit-participant | 57.9 | 49.2, 66.2 | 17.0 | 2.24 |
| Set-to-Failure | 42.4 | 35.3, 49.9 | 14.6 | 1.92 |
| Closest-VL | 52.9 | 45.4, 60.3 | 14.9 | 1.96 |
| Repeat-Binary | 57.3 | 50.4, 65.1 | 14.7 | 1.93 |
| Repeat-Continuous | 55.8 | 53.5, 61.1 | 7.6 | REF |
| ***40+*** |  |  |  |  |
| Omit-participant | 61.5 | 56.3, 66.5 | 10.2 | 2.37 |
| Set-to-Failure | 49.0 | 44.3, 53.6 | 9.3 | 2.16 |
| Closest-VL | 57.4 | 52.7, 61.9 | 9.2 | 2.14 |
| Repeat-Binary | 59.3 | 55.3, 63.6 | 8.3 | 1.93 |
| Repeat-Continuous | 57.8 | 55.8, 60.1 | 4.3 | REF |
| **Race** |  |  |  |  |
| ***White*** |  |  |  |  |
| Omit-participant | 70.7 | 57.8, 80.9 | 23.1 | 2.66 |
| Set-to-Failure | 51.3 | 40.4, 62.0 | 21.6 | 2.48 |
| Closest-VL | 63.8 | 52.7, 73.5 | 20.8 | 2.39 |
| Repeat-Binary | 79.3 | 70.1, 89.6 | 19.5 | 2.24 |
| Repeat-Continuous | 77.5 | 71.3, 80.0 | 8.7 | REF |
| ***Black*** |  |  |  |  |
| Omit-participant | 55.0 | 50.2, 59.7 | 9.5 | 2.16 |
| Set-to-Failure | 42.9 | 38.7, 47.2 | 8.5 | 1.93 |
| Closest-VL | 51.2 | 47.0, 55.5 | 8.5 | 1.93 |
| Repeat-Binary | 57.9 | 54.0, 62.2 | 8.2 | 1.86 |
| Repeat-Continuous | 51.4 | 50.3, 54.7 | 4.4 | REF |
| ***Hispanic*** |  |  |  |  |
| Omit-participant | 68.1 | 56.3, 78.0 | 21.7 | 2.01 |
| Set-to-Failure | 51.1 | 41.0, 61.1 | 20.1 | 1.86 |
| Closest-VL | 62.0 | 51.7, 71.3 | 19.6 | 1.81 |
| Repeat-Binary | 68.1 | 58.9, 78.7 | 19.8 | 1.83 |
| Repeat-Continuous | 69.6 | 60.9, 71.7 | 10.8 | REF |
| ***Other*** |  |  |  |  |
| Omit-participant | 76.9 | 47.9, 92.4 | 44.5 | 3.11 |
| Set-to-Failure | 47.6 | 27.9, 68.2 | 40.3 | 2.82 |
| Closest-VL | 57.1 | 36.0, 76.0 | 40.0 | 2.80 |
| Repeat-Binary | 71.5 | 57.6, 88.7 | 31.1 | 2.17 |
| Repeat-Continuous | 71.4 | 61.9, 76.2 | 14.3 | REF |

**Supplemental Table 2.** The estimated viral suppression risk ratio (RR), RR 95% CI, RR 95% CI width, and CI width ratio at month 24 using the SMVL (omit-participant, set-to-failure, closest-VL) and RMVL (repeat-binary, repeat-continuous) frameworks.

| **Characteristic** | **Risk Ratio (RR)** | **RR 95% CI** | **CI Width** | **CI Width ratio** |
| --- | --- | --- | --- | --- |
| **Age** |  |  |  |  |
| ***30-39 vs. 18-29*** |  |  |  |  |
| Omit-participant | 1.21 | 0.91, 1.60 | 0.69 | 3.83 |
| Set-to-Failure | 1.29 | 0.93, 1.77 | 0.84 | 4.67 |
| Closest-VL | 1.22 | 0.94, 1.58 | 0.64 | 3.56 |
| Repeat-Binary | 0.999 | 0.82, 1.22 | 0.40 | 2.22 |
| Repeat-Continuous | 1.02 | 0.96, 1.14 | 0.18 | REF |
| ***40+ vs. 18-29*** |  |  |  |  |
| Omit-participant | 1.28 | 0.997, 1.65 | 0.65 | 3.84 |
| Set-to-Failure | 1.48 | 1.11, 1.98 | 0.87 | 5.12 |
| Closest-VL | 1.32 | 1.05, 1.67 | 0.62 | 3.65 |
| Repeat-Binary | 1.03 | 0.87, 1.23 | 0.36 | 2.12 |
| Repeat-Continuous | 1.06 | 0.97, 1.14 | 0.17 | REF |
|  |  |  |  |  |
| **Race** |  |  |  |  |
| ***Black vs. White*** |  |  |  |  |
| Omit-participant | 0.78 | 0.65, 0.94 | 0.29 | 2.64 |
| Set-to-Failure | 0.84 | 0.66, 1.06 | 0.40 | 3.64 |
| Closest-VL | 0.80 | 0.67, 0.97 | 0.30 | 2.73 |
| Repeat-Binary | 0.73 | 0.63, 0.84 | 0.21 | 1.91 |
| Repeat-Continuous | 0.66 | 0.64, 0.75 | 0.11 | REF |
| ***Hispanic vs. White*** |  |  |  |  |
| Omit-participant | 1.09 | 0.77, 1.53 | 0.76 | 4.22 |
| Set-to-Failure | 1.00 | 0.74, 1.34 | 0.60 | 3.33 |
| Closest-VL | 0.97 | 0.77, 1.22 | 0.45 | 2.50 |
| Repeat-Binary | 0.86 | 0.71, 1.04 | 0.33 | 1.83 |
| Repeat-Continuous | 0.90 | 0.80, 0.98 | 0.18 | REF |
| ***Other vs. White*** |  |  |  |  |
| Omit-participant | 0.96 | 0.76, 1.21 | 0.45 | 1.80 |
| Set-to-Failure | 0.93 | 0.57, 1.53 | 0.96 | 3.84 |
| Closest-VL | 0.90 | 0.60, 1.34 | 0.74 | 2.96 |
| Repeat-Binary | 0.90 | 0.70, 1.16 | 0.46 | 1.84 |
| Repeat-Continuous | 0.92 | 0.80, 1.05 | 0.25 | REF |
