# Supplementary figures and images for: A Comparison of Methods for Analyzing Viral Load Data in Studies of HIV Patients
Source: PLoS One. 2015 Jun 19;10(6):e0130090. doi: 10.1371/journal.pone.0130090 (PMC4474923; doi:10.1371/journal.pone.0130090)

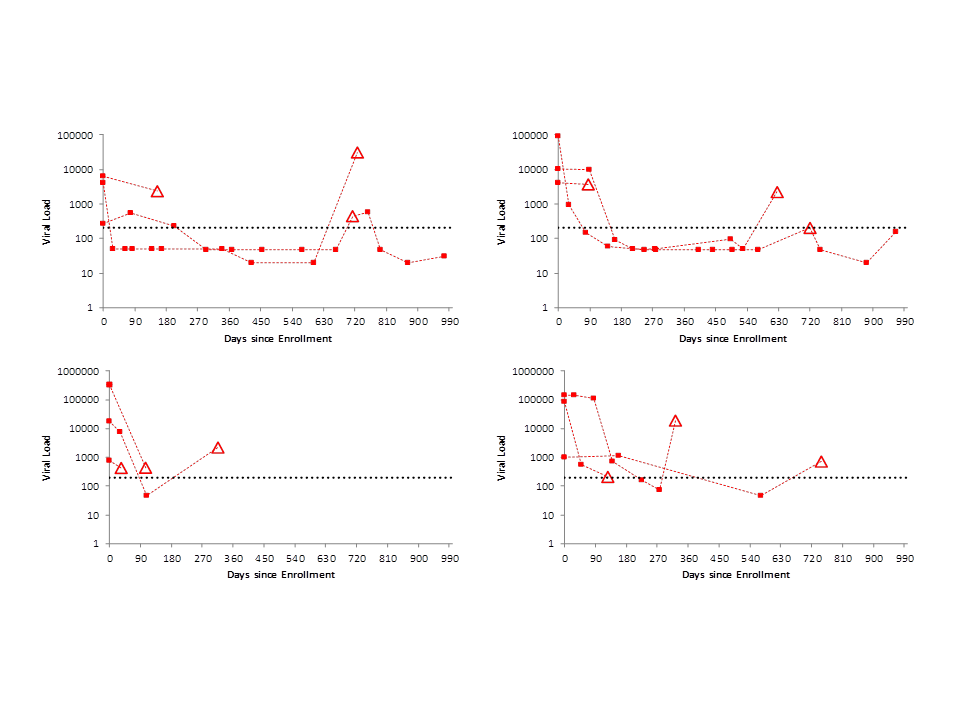

Supplement: S1 Fig — The 12 aged 18–29 participants for the three SMVL framework analyses (omit-participant, set-to-failure, closest-VL) that are analyzed as non-suppressed or set to missing and are predicted as suppressed using the RMVL repeat-continuous model at month 24. The triangles represent values used for the SMVL analyses and the “window” is from 540–780 days. Triangles not within the “window” are set to missing (omit-participant) or failure (set-to-failure) or non-suppressed (closest-VL method). (TIF) [file pone.0130090.s002.tif]
