# Supplementary material for: A Comparison of Methods for Analyzing Viral Load Data in Studies of HIV Patients
Source: PLoS One. 2015 Jun 19;10(6):e0130090. doi: 10.1371/journal.pone.0130090 (PMC4474923; doi:10.1371/journal.pone.0130090)
Supplement: S1 Table — The percent of the RIC study participants with virologic suppression, percent 95% CI, 95% CI width, and CI width ratio at month 24 using the SMVL (omit-participant, set-to-failure, closest-VL) and RMVL (repeat-binary, repeat-continuous) frameworks. (DOCX) [file pone.0130090.s003.docx]

**S1 Table.** The percent of the RIC study participants with virologic suppression, percent 95% CI, 95% CI width, and CI width ratio at month 24 using the SMVL (omit-participant, set-to-failure, closest-VL) and RMVL (repeat-binary, repeat-continuous) frameworks.

| **Characteristic** | **Percent Suppressed (VL <200)** | **Percent 95% CI** | **Percent 95% CI Width** | **CI Width Ratio** |
| --- | --- | --- | --- | --- |
| **Overall** |  |  |  |  |
| Omit-participant | 58.8 | 54.8, 63.1 | 8.23 | 2.12 |
| Set-to-Failure | 45.0 | 41.5, 48.8 | 7.27 | 1.87 |
| Closest-VL | 54.2 | 50.7, 57.9 | 7.28 | 1.87 |
| Repeat-Binary | 58.5 | 55.2, 61.9 | 6.69 | 1.72 |
| Repeat-Continuous | 56.7 | 55.3, 59.2 | 3.89 | REF |
| **Age** |  |  |  |  |
| ***18 - 29*** |  |  |  |  |
| Omit-participant | 47.9 | 36.8, 59.3 | 22.5 | 2.96 |
| Set-to-Failure | 33.0 | 24.8, 42.5 | 17.7 | 2.33 |
| Closest-VL | 43.4 | 34.3, 53.0 | 18.7 | 2.46 |
| Repeat-Binary | 57.4 | 49.0, 67.1 | 18.1 | 2.38 |
| Repeat-Continuous | 54.7 | 50.9, 58.5 | 7.6 | REF |
| ***30-39*** |  |  |  |  |
| Omit-participant | 57.9 | 49.2, 66.2 | 17.0 | 2.24 |
| Set-to-Failure | 42.4 | 35.3, 49.9 | 14.6 | 1.92 |
| Closest-VL | 52.9 | 45.4, 60.3 | 14.9 | 1.96 |
| Repeat-Binary | 57.3 | 50.4, 65.1 | 14.7 | 1.93 |
| Repeat-Continuous | 55.8 | 53.5, 61.1 | 7.6 | REF |
| ***40+*** |  |  |  |  |
| Omit-participant | 61.5 | 56.3, 66.5 | 10.2 | 2.37 |
| Set-to-Failure | 49.0 | 44.3, 53.6 | 9.3 | 2.16 |
| Closest-VL | 57.4 | 52.7, 61.9 | 9.2 | 2.14 |
| Repeat-Binary | 59.3 | 55.3, 63.6 | 8.3 | 1.93 |
| Repeat-Continuous | 57.8 | 55.8, 60.1 | 4.3 | REF |
| **Race** |  |  |  |  |
| ***White*** |  |  |  |  |
| Omit-participant | 70.7 | 57.8, 80.9 | 23.1 | 2.66 |
| Set-to-Failure | 51.3 | 40.4, 62.0 | 21.6 | 2.48 |
| Closest-VL | 63.8 | 52.7, 73.5 | 20.8 | 2.39 |
| Repeat-Binary | 79.3 | 70.1, 89.6 | 19.5 | 2.24 |
| Repeat-Continuous | 77.5 | 71.3, 80.0 | 8.7 | REF |
| ***Black*** |  |  |  |  |
| Omit-participant | 55.0 | 50.2, 59.7 | 9.5 | 2.16 |
| Set-to-Failure | 42.9 | 38.7, 47.2 | 8.5 | 1.93 |
| Closest-VL | 51.2 | 47.0, 55.5 | 8.5 | 1.93 |
| Repeat-Binary | 57.9 | 54.0, 62.2 | 8.2 | 1.86 |
| Repeat-Continuous | 51.4 | 50.3, 54.7 | 4.4 | REF |
| ***Hispanic*** |  |  |  |  |
| Omit-participant | 68.1 | 56.3, 78.0 | 21.7 | 2.01 |
| Set-to-Failure | 51.1 | 41.0, 61.1 | 20.1 | 1.86 |
| Closest-VL | 62.0 | 51.7, 71.3 | 19.6 | 1.81 |
| Repeat-Binary | 68.1 | 58.9, 78.7 | 19.8 | 1.83 |
| Repeat-Continuous | 69.6 | 60.9, 71.7 | 10.8 | REF |
| ***Other*** |  |  |  |  |
| Omit-participant | 76.9 | 47.9, 92.4 | 44.5 | 3.11 |
| Set-to-Failure | 47.6 | 27.9, 68.2 | 40.3 | 2.82 |
| Closest-VL | 57.1 | 36.0, 76.0 | 40.0 | 2.80 |
| Repeat-Binary | 71.5 | 57.6, 88.7 | 31.1 | 2.17 |
| Repeat-Continuous | 71.4 | 61.9, 76.2 | 14.3 | REF |
