# Supplementary material for: A Comparison of Methods for Analyzing Viral Load Data in Studies of HIV Patients
Source: PLoS One. 2015 Jun 19;10(6):e0130090. doi: 10.1371/journal.pone.0130090 (PMC4474923; doi:10.1371/journal.pone.0130090)
Supplement: S2 Table — The estimated viral suppression risk ratio (RR), RR 95% CI, RR 95% CI width, and CI width ratio at month 24 using the SMVL (omit-participant, set-to-failure, closest-VL) and RMVL (repeat-binary, repeat-continuous) frameworks. (DOCX) [file pone.0130090.s004.docx]

**S2 Table.** The estimated viral suppression risk ratio (RR), RR 95% CI, RR 95% CI width, and CI width ratio at month 24 using the SMVL (omit-participant, set-to-failure, closest-VL) and RMVL (repeat-binary, repeat-continuous) frameworks.

| **Characteristic** | **Risk Ratio (RR)** | **RR 95% CI** | **CI Width** | **CI Width ratio** |
| --- | --- | --- | --- | --- |
| **Age** |  |  |  |  |
| ***30-39 vs. 18-29*** |  |  |  |  |
| Omit-participant | 1.21 | 0.91, 1.60 | 0.69 | 3.83 |
| Set-to-Failure | 1.29 | 0.93, 1.77 | 0.84 | 4.67 |
| Closest-VL | 1.22 | 0.94, 1.58 | 0.64 | 3.56 |
| Repeat-Binary | 0.999 | 0.82, 1.22 | 0.40 | 2.22 |
| Repeat-Continuous | 1.02 | 0.96, 1.14 | 0.18 | REF |
| ***40+ vs. 18-29*** |  |  |  |  |
| Omit-participant | 1.28 | 0.997, 1.65 | 0.65 | 3.84 |
| Set-to-Failure | 1.48 | 1.11, 1.98 | 0.87 | 5.12 |
| Closest-VL | 1.32 | 1.05, 1.67 | 0.62 | 3.65 |
| Repeat-Binary | 1.03 | 0.87, 1.23 | 0.36 | 2.12 |
| Repeat-Continuous | 1.06 | 0.97, 1.14 | 0.17 | REF |
|  |  |  |  |  |
| **Race** |  |  |  |  |
| ***Black vs. White*** |  |  |  |  |
| Omit-participant | 0.78 | 0.65, 0.94 | 0.29 | 2.64 |
| Set-to-Failure | 0.84 | 0.66, 1.06 | 0.40 | 3.64 |
| Closest-VL | 0.80 | 0.67, 0.97 | 0.30 | 2.73 |
| Repeat-Binary | 0.73 | 0.63, 0.84 | 0.21 | 1.91 |
| Repeat-Continuous | 0.66 | 0.64, 0.75 | 0.11 | REF |
| ***Hispanic vs. White*** |  |  |  |  |
| Omit-participant | 1.09 | 0.77, 1.53 | 0.76 | 4.22 |
| Set-to-Failure | 1.00 | 0.74, 1.34 | 0.60 | 3.33 |
| Closest-VL | 0.97 | 0.77, 1.22 | 0.45 | 2.50 |
| Repeat-Binary | 0.86 | 0.71, 1.04 | 0.33 | 1.83 |
| Repeat-Continuous | 0.90 | 0.80, 0.98 | 0.18 | REF |
| ***Other vs. White*** |  |  |  |  |
| Omit-participant | 0.96 | 0.76, 1.21 | 0.45 | 1.80 |
| Set-to-Failure | 0.93 | 0.57, 1.53 | 0.96 | 3.84 |
| Closest-VL | 0.90 | 0.60, 1.34 | 0.74 | 2.96 |
| Repeat-Binary | 0.90 | 0.70, 1.16 | 0.46 | 1.84 |
| Repeat-Continuous | 0.92 | 0.80, 1.05 | 0.25 | REF |
